# Lectins, Interconnecting Proteins with Biotechnological/Pharmacological and Therapeutic Applications

**DOI:** 10.1155/2017/1594074

**Published:** 2017-03-07

**Authors:** Luana Cassandra Breitenbach Barroso Coelho, Priscila Marcelino dos Santos Silva, Vera Lúcia de Menezes Lima, Emmanuel Viana Pontual, Patrícia Maria Guedes Paiva, Thiago Henrique Napoleão, Maria Tereza dos Santos Correia

**Affiliations:** ^1^Departamento de Bioquímica, Centro de Biociências, Universidade Federal de Pernambuco, Av. Prof. Moraes Rego 1235, Cidade Universitária, 50.670-901 Recife, PE, Brazil; ^2^Departamento de Morfologia e Fisiologia Animal, Universidade Federal Rural de Pernambuco, Rua Dom Manuel de Medeiros, s/n, Dois Irmãos, 52171-900 Recife, PE, Brazil

## Abstract

Lectins are proteins extensively used in biomedical applications with property to recognize carbohydrates through carbohydrate-binding sites, which identify glycans attached to cell surfaces, glycoconjugates, or free sugars, detecting abnormal cells and biomarkers related to diseases. These lectin abilities promoted interesting results in experimental treatments of immunological diseases, wounds, and cancer. Lectins obtained from virus, microorganisms, algae, animals, and plants were reported as modulators and tool markers in vivo and in vitro; these molecules also play a role in the induction of mitosis and immune responses, contributing for resolution of infections and inflammations. Lectins revealed healing effect through induction of reepithelialization and cicatrization of wounds. Some lectins have been efficient agents against virus, fungi, bacteria, and helminths at low concentrations. Lectin-mediated bioadhesion has been an interesting characteristic for development of drug delivery systems. Lectin histochemistry and lectin-based biosensors are useful to detect transformed tissues and biomarkers related to disease occurrence; antitumor lectins reported are promising for cancer therapy. Here, we address lectins from distinct sources with some biological effect and biotechnological potential in the diagnosis and therapeutic of diseases, highlighting many advances in this growing field.

## 1. Introduction

Considering the diverse diseases and infectious agents that affect the human species and their consequences, the biotechnological field has searched biorecognition molecules from natural or recombinant sources with diagnostic and therapeutic potential. The key for efficient detection, treatment, and healing of pathological conditions is the biorecognition event. The identification of carbohydrate moieties in cell surface and glycoconjugates has been performed with the use of lectins, a heterogeneous group of proteins (or glycoproteins) of nonimmune origin that bind carbohydrates through molecular sites, with high affinity and specificity [1]. These particular lectin sites interact with mono- or oligosaccharides through no covalent linkage involving hydrogen bonds, van der Walls and hydrophobic interactions, with reversibility, high specificity, and no catalytic or immune activity [2, 3]. Lectins have been isolated from distinct sources such as viruses, bacteria, fungi, algae, animals, and plants [1]; they show specificity to distinct carbohydrates, such as mannose, sialic acid, fucose, N-acetylglucosamine, galactose/N-acetylgalactosamine, complex glycans, and glycoproteins [4, 5]. Lectins recognize carbohydrates and glycoconjugates in cells, tissue sections, and biological fluids, being valuable tools in biotechnology, including diagnosis, and pharmacological and therapeutic applications [6–8].

Some lectins mediate the infection mechanism through the interaction of viral lectins with glycan chains on surface of host cells, resulting in the virus entrance into cell [30]. Sialic acid-specific lectins such as influenza virus hemagglutinin were used to search antiviral drugs and inhibitors that can remove or block the sialic acid site in host cells in order to prevent the binding [31]. Lectins from bacteria play a role in the bacterial virulence through the binding between bacterial lectins and specific carbohydrate moieties of host cells, being an important factor for recognition and adhesion [32, 33]. Strong anti-HIV (human immunodeficiency virus) activity in vitro has been described to bacterial lectins [34–36]. Various fungal lectins distributed among mushrooms, microfungi, and yeasts exhibited many physiological effects and biomedical applications. Similar to bacterial lectins, the interaction between fungal lectins and host glycans has an important role in the infection process of fungi [37]. Fungal lectins revealed immunomodulatory, mitogenic, antiproliferative, antitumoral, antiviral, and antimicrobial activities [5, 38].

A large number of algal lectins have also attracted considerable attention for biomedical applications, including anti-HIV, antitumoral, antimicrobial, anti-inflammatory, and antinociceptive activities [39]. Animal lectins play a role in various physiological processes, such as metastasis of cancer, apoptosis pathways, and immunomodulation [40, 41]. Many lectins isolated from animal tissues were investigated as apoptotic agents, immunomodulatory, antiviral therapy, and anticancer drug targets [41, 42]. Plant lectins purified and characterized from distinct plant families and tissues including seeds, barks, leaves, roots, fruits, and flowers exhibited various molecular features, structures, and carbohydrate specificities [43]. The Leguminosae family has the largest group of well-characterized legume lectins, which are interesting due to a variety of carbohydrate specificity and greater availability in nature [44]. In general, a wide range of biological applications has been attributed to plant lectins, such as mediators of inflammatory and immune response; antiviral, antibacterial, antifungal, and antihelminthic agents, healing effect, drug delivery, histochemical markers, biosensing of diseases, and antitumoral activities [3, 45, 46]. This review discusses representative lectins from distinct natural sources, highlighting some biological effects in vivo and in vitro and their potential for diagnosis and therapeutic applications.

## 2. Lectin Induced Mechanisms of Immunological and Inflammatory Responses

Immunological and inflammatory responses play a role in the protection of an organism against an invasive agent and transformed cells. The immune system acts through two ways known as innate and adaptive immune responses, activated by a group of cells and molecules that promote the inactivation or destruction of aggressive agent [47]. There are neutrophils, eosinophils, basophils, and monocytes/macrophages, with specific functions and ability to produce and release molecules named cytokines, which modulate the activation of immune cells, inflammation, and humoral response. The search for biomolecules that can modulate (up or down) mechanisms of immune response is attractive for adjustment of immune conditions and therapeutic applications in diverse immune response-related diseases and infections.

**Figure 1:**
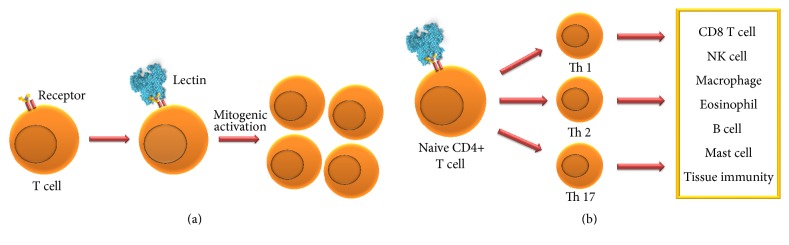
Lectins induced in vitro mitogenic activation of T cells (a) and stimulated in vivo Th1, Th2, and Th17 responses (b).

Several lectins from distinct sources showed immunomodulatory effects, such as mitogenic activity (Figure 1(a)) and induction of T helper type 1 (Th1), type 2 (Th2), or type 17 (Th17) responses (Figure 1(b)). The pivotal step to start immunomodulatory activities of lectins is the binding of lectins to glycans targets on cell surface, which have the role of lectin-receptors [48]. Lectin binding can induce the immune response through mediators, such as second messengers released from the membrane. For example, diacylglycerol and inositol 1,4,5-triphosphate generated through the hydrolysis of phosphatidylinositol 4,5-diphosphate; increase in cytosolic Ca^2+^ levels; release of cytokines specifics; binding to receptors distributed in stimulatory regions or domains; cascade started by linkage to TCR receptor; and further other mechanisms incompletely elucidated [49, 50].

Fungal lectins have been highlighted as potent modulators of immune response. TML-1 and TML-2 are lectins from the mushroom* Tricholoma mongolicum*, which induced the Th1 response through the activation of macrophages from lectin-treated mice, and stimulated the production of nitrite ion and cytokine tumor necrosis factor (TNF) by macrophages. Additionally, both lectins promoted in vivo activation of mouse macrophages, triggering the inhibition of tumor cell growth [51]. These effects may result from the lectin binding to the glycan determinants on mouse macrophages surface, modulating the immune response. A lectin purified from the edible mushroom* Volvariella volvacea*, named VVL, induced the proliferation of murine splenic lymphocytes and stimulated the transcriptional expression of interleukin 2 (IL-2) and interferon *γ* (INF*γ*) in vitro, stimulating the Th1 response [52]. Similar activity was evidenced by a mushroom lectin from* Ganoderma capense*, which induced high proliferative response in mouse splenocytes [53]. The lectin isolated from another edible mushroom* Pleurotus citrinopileatus* showed in vitro mitogenic activity on mouse splenocytes, with the maximal stimulation at 2 *µ*M of lectin concentration [54]. These lectins could have some similarity with fungal immunomodulatory proteins, such as Fip-vvo, in the slight N-terminal sequence, which may be related to this proliferative response [52, 53].

Immunomodulatory activities of* Agaricus bisporus* lectin (ABL) from edible mushroom acted on innate and adaptive immune responses in vivo and in vitro but showed an inhibitory and antiproliferative effect on macrophages. ABL downregulated in vitro nitric oxide (NO) production by mouse peritoneal macrophages after lipopolysaccharide stimuli and inhibited mononuclear cell proliferation under different conditions. Orally administered ABL to BALB/c mice also induced inhibition of NO production by peritoneal macrophages and stimulated a tumor development. The interaction of ABL-macrophages occurs through the terminal residues of T antigen and sialyl-T antigen in mucin-type O-glycans exposed on macrophages, and this interaction can modify the Akt signaling pathway. ABL binding can block Akt phosphorylation, resulting in the inhibition of NO production, and cytokine production by macrophages [55]. Unlike this, the oral treatment with ABL promoted a reduction of the tumor growth and attenuated symptoms of experimental autoimmune encephalomyelitis in Wistar rats [55].

A mycelial lectin from* Aspergillus nidulans *microfungi induced an increase of NO production, and IFN*γ* and IL-6 levels also enhanced in splenocyte cultures from treated Swiss albino mice groups. Additionally, the lectin promoted an antianaphylactic effect and prevented Arthus reaction in vivo; a therapeutic potential against ulcerative colitis was observed in rats pretreated with the lectin via intraperitoneal injection that showed better recovery comparing with posttreated rats. The high specificity of this lectin for N-acetylgalactosamine (GalNAc) residues on epithelial cells could trigger the therapeutic effect [56].

Seaweed lectins are widely studied due to attractive biological activities. The mitogenic activity for T lymphocytes from mouse spleen was firstly reported to a lectin from the red seaweed* Carpopeltis flabellate* (Carnin) [57]. The lectin from green seaweed* Caulerpa cupressoides* (CcL) showed antinociceptive and anti-inflammatory activities on zymosan-induced arthritis in rat temporomandibular joint. Rats treated with CcL reduced until 89.5% zymosan-induced hypernociception and leukocyte influx until 98.5%. The lectin also lowers the expression levels of IL-1*β* and TNF*α* compared with nontreated group, being a probable way for antinociception and anti-inflammation effects lectin induced [58].

Animal lectins present in different tissues from insects, fishes, and mammalians, among others, play important roles in immune system regulation, being applied as potent immunomodulators. Mitogenic activity was reported to a D-galactose-binding lectin from* Musca domestica* pupae. Proliferation of mouse splenocytes was stimulated in the presence of the lectin in vitro with maximal activity at a concentration of 20 *µ*g/mL [59]. A rhamnose-specific lectin isolated from ovaries of the grass carp fish (*Ctenopharyngodon idellus*) showed immunostimulatory effect, including proliferative response in murine splenocytes and peritoneal exudates cells. Grass carp lectin also induced IL-2 and IFN*γ* expression in treated splenocytes [60]. A lectin also was isolated from roe of the grass carp and similarly to lectin from grass carp ovaries showed mitogenic activity on murine splenocytes and stimulated the phagocytic activity of sea bream macrophages [61]. A mannose-binding lectin from the ovary of cobia fish (*Rachycentron canadum*) also exhibited mitogenic activity on mouse splenocytes, at 14 *µ*M [62]. Another mannose-binding lectin, isolated from serum of cobia named* Rachycentron canadum* lectin (RcaL), was also reported as immunomodulatory agent. The mitogenic response and cytokine production in splenocytes from mice in vitro treated with RcaL were evaluated. A high proliferation index to treated cells and induction of high levels of IL-2 and IL-6 production were observed; RcaL was a potential mitogenic agent [63]. RcaL induced Th 1 response in treated cultures of mice splenocytes through IFN-*γ* and NO production without cytotoxicity [64]. A mannose-specific lectin from serum of* Oreochromis niloticus* (Nile tilapia fish) denominated OniL demonstrated in vitro Th1 response induction on mice splenocytes. OniL also stimulated high levels of IFN*γ* production and low levels of IL-10 and nitrite, without cytotoxic effect [65].

Galectins are a family of *β*-galactoside-binding lectins occurring in animals and play important roles in diverse immunomodulatory processes. Galectin-1 revealed a regulatory role in the thymocyte maturation through the interaction with O-glycans on immature cortical thymocyte surface [66]. In addition, galectin-1 induced apoptosis of immature thymocytes by activation of a p53 pathway [67] and inhibited cell growth, as observed to human leukemia T cells [68]. It is suggested that galectin-1 induced apoptosis through the regulation of intracellular signals, such as activation of AP-1 transcription factor, downregulation of Bcl-2, and activation of caspases [69]. Galectin-3 behaved as a mitogenic and antiapoptotic agent on human leukemia T cells with apoptosis induced by Fas receptor ligation and by staurosporine [70]. The high sequence similarity of galectin-3 with an antiapoptotic protein family named Bcl-2, including the presence of the NWGR motif, highly conserved in Bcl-2, suggests that galectin-3 can modulate the Bcl-2 pathway and inhibit apoptosis [70]. Galectin-3 also prevents the apoptosis by protection of the mitochondrial membrane and inhibition of reactive oxygen species production [69]. Moreover, galectin-3 acts as a chemotactic agent to monocytes and macrophages by a G-protein pathway [69].

A group of C-type lectins known as macrophage galactose-type lectin (MGL) are commonly glycan-binding receptors on dendritic cells and macrophages from human immune system that participate in immune response steps, as pathogen recognition, endocytosis, and presentation of antigens to T cells. In the activation of human T cells, MGL recognize Tn antigens on the CD45 of effector T cells, triggering reduction in the phosphatase activity of CD45, inhibition of T cell proliferation and inflammatory cytokines production, and T cell apoptosis [71]. Human MGL can enhance IL-10 production by dendritic cells and induce the proliferation of regulatory T cells and CD8+ T cell responses [71].

Many mechanisms of immunomodulation have been attributed to plant lectins. Phytohemagglutinin (PHA) is an N-acetylgalactosamine specific lectin from red kidney bean (*Phaseolus vulgaris*) and one of the first lectins identified as mitogenic agent to lymphocytes [72]. Concanavalin A (Con A) is a mannose/glucose specific lectin isolated from* Canavalia ensiformis* seeds and is among some lectins used as models in lectin-carbohydrate interaction studies. Con A has been reported as mitogenic agent on CD4+ T cells and as antitumoral. Autophagic pathway induced by Con A is beginning when the lectin binds to the mannose residues on the cell membrane, internalized through clathrin-mediated endocytosis to the mitochondria, altering its membrane permeability and inducing the mitochondria autophagy; tumoral cells suffer the cell death [73]. Con A is commonly used as positive control in the evaluation of immunomodulation involving other lectins.

Immunomodulatory activity reported to Korean mistletoe (*Viscum album*) lectin (KML) showed a stimulatory effect on expression of cytokines IL-3, IL-23, and TNF*α* and in the intracellular reactive oxygen species (ROS) generation, while inhibiting events induced by lipopolysacharride, such as the production of IL-10 and NO [74]. Agglutinin from* Abrus precatorius *has been reported as an inductor of Th1 immune response through splenocyte activation and induction of IL-2, IFN*γ*, and TNF*αβ* cytokine production. Native and denatured forms of* A. precatorius* agglutinin [75] also induced NK-cell activation and thymocyte proliferation. ConBr, a mannose-binding lectin from* Canavalia brasiliensis* seeds, demonstrated mitogenic activity on in vitro splenocytes showed upregulation in the IL-2, IL-6, and IFN*γ* expression and a decrease in IL-10 expression [76].

Isoforms of mannose-binding lectins purified from* Cratylia mollis* leguminous seeds, Cramoll 1 and Cramoll 1,4, demonstrated high potential to stimulate human T lymphocyte mitogen in vitro [77]. Cramoll 1,4 and Con A also showed high mitogenic activity in vivo in splenocytes from mice previously inoculated and were potential inductors of cytokines IL-2, IL-6, and IFN*γ* release, as well as NO production, stimulating the Th1 response [78]. It is suggested that its mitogenic activity on T lymphocytes is induced by transmembrane signals started with the Cramoll binding to glycans of the cell surface, and the proliferation of splenocytes may be induced by a TCR-dependent mechanism [78]. Another study reported the potential of Cramoll 1,4 to stimulate rat spleen lymphocytes activation in vivo through a significant increase of cytosolic Ca^2+^ and ROS Cramoll 1,4-induced. In this case, the production of ROS may be induced by NADPH oxidase and to have relation with the increase of Ca^2+^ release. Cramoll 1,4 also promoted an increase in IL-1*β* levels and stimulated the Th2 response [79]. Immunomodulatory activity of Cramoll 1,4, Con A, and PHA was demonstrated in experimental cultures of treated mice lymphocytes by the induction of Th1 response, showing NO suppression, high production of IFN*γ*, low production of IL-10, and anti-inflammatory activity [80]. Cramoll 1,4 also stimulated cytokine releases in Th 17 pathway, inducing the production of IL-6, IL-17A, IL-22, and IL-23 as well as immunologic memory in cultured splenocytes [81]. Immunomodulatory effects of Cramoll 1,4 and recombinant Cramoll (rCramoll) were observed on cell culture of mice peritoneal exudates infected and noninfected with* Staphylococcus aureus*. The lectins induced the production of IL-1*β* and IFN*γ*, reducing the expression of TNF*α* and IL-6 in* S. aureus* infected cells. NO and ROS production enhanced, and phagocytic activity of* S. aureus *increased. Both lectins stimulate phagocytic activity and production of proinflammatory cytokines by activation of intracellular signaling cascades [82].

A lectin purified from taro (*Colocasia esculenta*), the tarin, promoted proliferation of mouse splenocytes in vitro and in vivo. Optimum in vitro cellular proliferation was observed in mouse splenocytes treated with 500 ng of tarin and total in vivo proliferation was 3.3-fold higher than control group [83]. A lectin from* Ziziphus oenoplia *Rhamnaceae,* Z. oenoplia* seed lectin (ZOSL), showed potential antiallergic and anti-inflammatory effects by prevention of Arthus reaction and anaphylactic shock in vivo in Wistar albino rats after oral administration of ZOSL (200 mg/kg of b.w.) [84].

Some lectins isolated from edible tissues of plants have immunomodulatory activity. Garlic (*Allium sativum*) lectins ASA I and ASA II revealed a mitogenic effect in human peripheral blood lymphocytes, murine splenocytes, and thymocytes and stimulated in vitro histamine release from leukocytes in atopic patients when compared with nonatopic individuals [85]. A mannose-specific lectin from onion (*Allium cepa* agglutinin, ACA) also showed mitogenic activity in murine thymocytes, as well as a high production of IFN*γ* and IL-2. An increase in the release of NO and in the production of cytokines TNF*α* and IL-12 by murine macrophage cell line (RAW264.7) and rat peritoneal macrophages was observed after 24 h of the lectin treatment, showing an inductor effect on Th1-type immune response, in vitro [86]. Banana (*Musa acuminata*) lectin (BanLec) showed in vivo immunomodulatory effect. BanLec was orally administered to mice and after seven days, mouse peripheral blood showed an increased level in IL-10, IL-17, and TNF*α* and a reduction of IL-6 and IFN*γ*. In addition, CD4+ T cells enhanced while the CD8+ T cell population reduced in mouse thymus [87].

These findings show the importance of lectin-based recognitions to regulate inflammatory processes and immune responses with their potential for biotechnological applications, to understand immune mechanisms and therapeutic tools for immunological disorders and diseases.

## 3. Antifungal and Antiparasitic Activities of Lectins

Recent studies have demonstrated the potential of lectins from different origin and carbohydrate specificities as antifungal and antiparasitic agents. Plant lectins investigated for antifungal potential, mainly against phytopathogenic species, have most reported antifungal effects binding to hyphae, causing inhibition of growth and prevention of spore germination. For example, a lectin isolated from* Myracrodruon urundeuva *heartwood was able to inhibit in more than 50% the mycelial growth of* Fusarium oxysporum*,* F. decemcellulare*, and* F. fusarioides* [26].* Fusarium* growth was also impaired by a galactose-specific lectin isolated from* Bauhinia monandra* secondary roots, with highest effect (30% inhibition) on* F. solani *[45]. Inhibitory effects of lectins on growth of phytopathogens from other genera were also reported. Lectins from* Phaseolus vulgaris* seeds inhibited the growth of* Coprinus comatus *and* Rhizoctonia solani *[88] as well as* Valsa mali* [89]. A mannan-specific lectin from* Ophioglossum pedunculosum *roots strongly damaged the growth of* Sclerotium rolfsii* at 40 *μ*g/mL [90].

*Microgramma vacciniifolia* rhizome lectin had its carbohydrate-binding ability inhibited by glycosylated molecules from* F. oxysporum f. *sp.* lycopersici *mycelia [91]. These authors then suggested that the interaction between this lectin and fungal carbohydrates might be involved in the growth inhibitory property detected against races 1, 2, and 3 of this phytopathogen. However, the carbohydrate-binding sites of lectins are not always involved in the antifungal action. An example is the lectin from* Astragalus mongholicus* roots, which was active against* F. oxysporum*,* Colletotrichum *sp.,* Drechslera turcia*, and mainly* Botrytis cinerea*. The addition of lactose and galactose (inhibitors of hemagglutinating activity of this lectin) in the assay did not interfere with antifungal effect [92].

A chitin-binding lectin from* Setcreasea purpurea* rhizome inhibited the germination of* R. solani*,* Sclerotinia sclerotiorum*,* Penicillium italicum,* and* Helminthosporium maydis *spores with minimal inhibitory concentrations ranging from 48.1 to 96.2 *µ*g/mL [93]. In the same way, jackin and frutackin, two chitin-binding lectins from the genus* Artocarpus*, inhibited the germination of* Fusarium moniliforme *spores at a concentration of 2.25 mg/mL [94]. The authors of both works also reported that these lectins impaired the development of hyphae; the mycelia formed were not able to produce spores, and the chitin-binding property is probably involved in the fungistatic action. Interestingly, the antifungal property of* S. purpurea* lectin remained even after heat-treatment of the protein at 75°C [93].

Antifungal lectins have specificity regarding fungal species; mannose/glucose-binding lectin from* Capsium frutescens* var.* fasciculatum* seeds was able to inhibit the spore germination and hyphal growth of* Aspergillus flavus *and* Fusarium moniliforme* but showed no effect on* F. graminearum*,* F. solani*,* Physalospora piricola,* and* B. cinerea* [95].

Some plant lectins were used in plant transgenic researches; this is the case of lunatin, a glycosylated and metal-dependent lectin isolated from* Phaseolus lunatus *seeds, which showed potent antifungal activity against* Sclerotium rolfsii*,* P. piricola*,* F. oxysporum,* and* B. cinerea* [96]. Another example is the lectin from rhizome of* Ophiopogon japonicus*, with antifungal activity against* Gibberella saubinetii* and* R. solani*, showing minimal inhibitory concentrations of 0.06 and 0.05 mg/mL, respectively. This lectin is able to interact with glycans containing mannose [Man-*α*(1,3:1,6)-mannotriose, Man-*α*(1,3)-Man, Man-*α*(1,6)-Man, Man-*α*(1,2)-Man, Me*α*-D-man, and D-mannose] and its carbohydrate-binding sites shown to be very structurally similar to those from monocot lectins [97]. Transgenic plants expressing antifungal lectins were already effectively developed and tested under laboratory conditions. Transgenic rice plants expressing a stable monomeric mutant variant of* Allium sativum* leaf lectin exhibited reduced sensitiveness to sheath blight disease caused by* R. solani*, in comparison with nontransformed plants [98].

Fungi that are human and animal pathogens are also affected by antifungal lectins.* Helianthus annuus* seed lectin inhibited the growth and altered membrane permeability of* Candida tropicalis*,* Candida parapsilosis*,* Candida albicans*, and* Pichia membranifaciens*. This protein was also able to induce the formation of pseudohyphae and the production of reactive oxygen species in* C. tropicalis *[99]. A lectin isolated from* Cladonia verticillaris *lichen was able to inhibit the growth (35%) of dermatophyte* Trichophyton mentagrophytes* [100]. Klafke et al. [101] evaluated the potential of Con A and* Abelmoschus esculentus*,* Mucuna pruriens,* and* Clitoria fairchildiana* lectins against* Candida albicans*,* C. tropicalis*,* C. parapsilosis*,* Cryptococcus gattii*,* Cryptococcus neoformans*,* Malassezia pachydermatis*,* Rhodotorula* sp., and* Trichosporon* sp.; however, only* C. parapsilosis* growth was inhibited by these lectins. Pinheiro et al. [102] reported antifungal effect of* Talisia esculenta* lectin on arthroconidial forms of the dermatophyte* Microsporum canis* obtained from hairs of infected animals. Authors proved that the antifungal mechanism involved the carbohydrate-binding sites of this protein.

Antifungal lectins obtained from other organisms, such as the mussel* Crenomytilus grayanus*, contain a lectin able to inhibit germination of conidia from several* Aspergillus* strains [103]. A galactose/*N*-acetylgalactosamine-specific lectin from the mussel* Mytilus trossulus* impaired conidia germination of species belonging to* Fusarium*,* Trichoderma*,* Haematonectria*,* Aspergillus, *and* Alternaria* genera [103, 104].

The relationship between structure and antifungal activity of lectins is also target of researches. The lectin from the plant* Pinellia ternata f*.* angustata* contains two domains called PTADOM1 and PTA-DOM2, each one with a mannose-binding site. This lectin as well as the two separate domains (expressed in* Escherichia coli*) showed antifungal activity toward the phytopathogens* Alternaria alternata *and* Bipolaris sorokiniana* and the dermatophyte* Curvularia lunata*. The whole lectin showed higher activity than two separated domains, an expected result, since both domains exert antifungal action [105].

The effects of lectins on human and animal parasites investigated under different approaches include the determination of parasiticidal action, prevention of infection, and study of the involvement from carbohydrate-receptor interactions on the infective process. The lectins Con A and ricin promoted tegumental damage (basal vacuolation and swelling of the basal membrane invaginations) in adult worms of* Schistosoma mansoni *(schistosomiasis causer), which were prevented when assays were performed in presence of carbohydrates that inhibit these lectins [106]. Con A,* Triticum vulgaris,* and* Glycine max *lectins interfered with migration pattern of* Strongyloides ratti* (rat threadworm) along a sodium chloride gradient, which indicates the involvement of carbohydrate moieties in the chemosensory activity of labial sensilla in this nematode [107]. Lectins from* Coprinopsis cinerea*,* Aleuria aurantia,* and* Laccaria bicolor* showed larvistatic effect on* Haemonchus contortus *(Barber's pole worm), resulting in arresting at L1 phase; the lectin from* Marasmius oreades* promoted larval death [108]. Cramoll 1,4 was evaluated for in vivo antihelminthic activity using mice infected with* S. mansoni*; the treatment with this lectin led to decrease in the number of excreted eggs, recovered adult worms, and liver granulomas [109].

Plant lectins prevented the infection of intestinal epithelium of* Psetta maxima* fish by the myxozoan* Enteromyxum scophthalmi*. Con A and* Glycine max* lectins inhibited attachment and invasion of* E. scophthalmi* to the intestinal epithelium; authors suggested that these lectins acted by blocking* N*-acetylgalactosamine, galactose, mannose, and/or glucose residues that are important in the interaction between the parasite and intestinal cells [22]. A lectin isolated from* Synadenium carinatum* latex reduced the infection of murine macrophages by* Leishmania amazonensis* [110]. Authors also reported that the lectin showed no cytotoxicity to mammalian host cells and that the macrophages treated with the lectin showed increased expression of cytokines IL-12, IL-1, and TNF*α*.

Lectins also evaluated the use in control of host and vectors from parasites and virus. The* Microgramma vacciniifolia *rhizome lectin at 100 *µ*g/mL was able to promote death of* Biomphalaria glabrata *(intermediate host of* S. mansoni*) embryos and adults; in addition, the snails treated with the lectin laid a few number of eggs, among which several showed malformations [111]. The lectins from* Cratylia floribunda* (CFL) and* Dioclea guianensis* (Dgui) were also able to promote death of* B. glabrata *adult snails [112]. Larvicidal activity of lectins from* Myracrodruon urundeuva *bark, heartwood, and leaves against the mosquito* Aedes aegypti *(vector of the virus that causes dengue fever, chikungunya, and zika virus fever) was reported [113, 114]. In addition, lectins isolated from whole seeds and seed cake of* Moringa oleifera *demonstrated larvicidal, ovicidal, and oviposition-stimulant effects on* A. aegypti*, being considered important candidates for using in control of mosquito population, including in traps for egg capture [115–118].

## 4. Lectins for Healing Applications

Many researchers have reported healing effect induced by lectins. The healing is the process of tissue repair after trauma, in which a monitored group of cells and molecules trigger ordered phases to result in anatomical and functional restoration of injured tissues [119]. The repair in response to injury includes sequential phases of hemostasis, inflammatory phase, tissue formation (proliferation), and remodeling of extracellular matrix (tissue maturation). Firstly, coagulation factors and platelets promote the blood coagulation in the damaged tissue. Inflammatory cells as neutrophils and macrophages phagocyte damaged cells and extracellular matrix; thus, a new tissue starts its regeneration and finally the scar formation [120]. The role of lectins as healing agents is not completely clear; however, lectins may influence the immune response, production of cytokines, inflammatory response, and cell antiproliferative effect during the healing process [8]. Lectins have promoted healing effect in cutaneous wounds and modification of scarring process, with great results and therapeutic potential.

Antiproliferative effect of an edible mushroom* Agaricus bisporus* lectin (ABL) in a model of wound healing was evaluated on human ocular fibroblasts in vitro in order to test the control of scar formation [121]. Ocular fibroblast proliferation was inhibited until 40% and collagen lattice contraction was completely abolished at 100 *µ*g/mL of ABL. These effects indicate the potential of ABL to modulate the healing process and scar formation in human ocular tissue. ABL may inhibit the proliferation by the influence of growth factors, as epidermal growth factor and insulin. It was also proposed that ABL is internalized after the linkage and accumulates around the nucleus where it may block nuclear localizing sequence- (NLS-) dependent uptake of protein into the nucleus [121, 122].

A lectin isolated from the marine red algae* Bryothamnion seaforthii *(BSL) demonstrated prohealing potential on skin wounds in mice [123]. Induced wounds in dorsal thoracic region of mice were submitted to topical treatment with BSL. During the treatment, BSL showed proinflammatory effect and stimulated reduction of the wound areas. After 7 and 12 days, treatment with BSL promoted the synthesis of collagen by fibroblasts and active presence of young skin annexes, promoting the restructuration of the luminal epithelium and an effective wound closure. Here, BSL plays an immunomodulatory effect on immune cells during inflammatory and proliferative phases, where BSL demonstrated stimulatory activity on the migration of polymorphonuclear cells to injured site, and activation of fibroblasts, resulting in the prohealing effect [123].

Galectins 3 and 7 play important role in reepithelialization of wounds, according to study conducted in models of mice corneal wound healing [124]. Significant reduction in reepithelialization of wounds was observed in galectin 3-deficient (gal3^−/−^) mice lineage when compared with wild-type lineage (gal3^+/+^). In the in vitro healing assay the presence of galectin-3 stimulated reepithelialization of cornea wounds in gal3^+/+^ lineage, but not in gal3^−/−^, probably due to its deficiency of galectin 7. On the other hand, galectin 7 induced reepithelialization of cornea wounds in gal3^−/−^ and gal3^+/+^. These results represent a potential application of gal3 and gal7 for treatment of wounds. Members of galectin family have showed possibility of mediating cell-matrix interactions, mainly galectin 3, which has been expressed in inflammatory cells and fibroblasts and located at sites of corneal epithelial cell-matrix adhesion and may stimulate cell-matrix interaction and cell migration in the wound healing process [124].

Mannose-binding lectin (MBL) replacement therapy was reported as a healing strategy of a radiation-induced chronic ulcer [125]. In this study, a patient with an insufficient level of MBL and a chronic radiation-induced ulcer after treatment of breast cancer was observed, which even after 15 months of conservative treatment and plastic surgery a satisfactory healing was not obtained. Thus, an experimental intravenous therapy with human plasma-derived MBL was carried out for 6 weeks; after the treatment, a complete healing was obtained. MBL is a component of innate immunity and its role in the elimination of microorganisms and modulation of immune response could contribute to wound healing.

D-mannose-binding lectin from* Artocarpus integrifolia* (jackfruit) seeds known as KM+ or artocarpin promoted wound healing on rabbit corneal epithelium [126]. Firstly, lesion of 6.0 mm diameter area was induced on the cornea of both eyes of experimental and control groups. The lesions of groups were treated with KM+ and buffer, respectively; the wound areas were monitored by fluorescein staining. KM+ enhanced the neutrophil influx into the wound area and may stimulate the production of cytokines, contributing for corneal epithelium healing [126].

Prohealing potential on skin wounds in mice was showed by the native lectin of orchid tree* Bauhinia variegata* (nBVL) and its recombinant isoform (rBVL-1) [127]. Dorsal skin wounds were induced surgically in mice, followed by topical lectin treatment for 12 days. Both lectins promoted wound closure in treated animals, and all skin layers were restructured. It is suggested that the prohealing effects of nBVL and rBVL-1 are due to stimulatory potential of these lectins for mitosis of resident cells such as macrophages and mast cells, triggering the release of cytokines and the recruitment of neutrophils into the wound area. These prohealing properties are attractive for therapy applications involving skin wounds.

**Figure 2:**
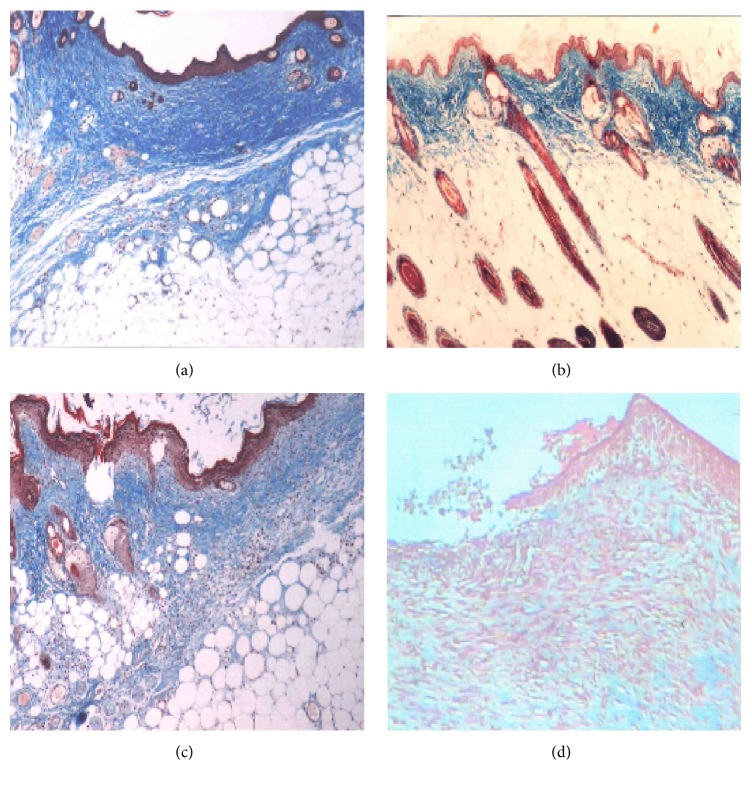
Histologic micrographs of wounds treated with Cramoll 1,4 and Con A in the 12th day in female albino Swiss mice. A total wound closure, reepithelialization, and deposition of collagen fibers are observed in Cramoll 1,4 treated group with 5 *µ*g/100 *µ*l (a), Cramoll 1,4 treated group with 10 *µ*g/100 *µ*l (b), and Con A (10 *µ*g/100 *µ*l) treated group (c). The formation of well-developed cutaneous annexes is present in Cramoll 1,4 (10 *µ*g/100 *µ*l) treated group (b). Incomplete reepithelialization without wound closure observed for control negative group administered with 150 mM NaCl (d).

Cramoll 1,4 has been reported as healing agent [8]. In order to analyze the cicatricial power, the lectin was applied to topical treatment of cutaneous wounds surgically induced in normal and immunocompromised albino Swiss mice [128]. Treated animals showed more edema formation and recruitment of more polymorphonuclear cells at the wounds. The prevalence of polymorphonuclear cells induced by Cramoll 1,4 is important to remove cell debris and microorganisms in the wound, favoring the healing [128]. Cramoll 1,4 also induced efficiently the granulation phase, collagen fiber deposition, and no incidence of microorganisms in all treated wounds, resulting in wound closure and repair faster than control groups. A parallel study conducted in female albino Swiss mice with skin wound surgically induced included daily treatment with Cramoll 1,4 (5 and 10 *µ*g/100 *µ*l) at 2nd, 7th, and 12th days. Macroscopic and histological aspects of treated groups were compared with a positive control group treated with Con A (10 *µ*g/100 *µ*l) and a negative control group administered with 150 mM NaCl. In the 12th day, wound closure, complete reepithelialization, a great deposition, and organization of collagen fibers and formation of cutaneous annexes were observed in wounds treated with lectins; no wound closure and partial reepithelialization were visualized in negative control group (Figure 2). Another study related the occurrence of gradual healing process induced by a hydrogel containing Cramoll 1,4 on experimental second-degree burns in rats [129]. On the 7th day of treatment, treated group showed higher edema, exudates, and necrosis. With more 7 days, tissue reepithelialization and moderate autolysis were observed. With more two weeks, tissue epithelialization was completed; and in the 35th day was observed a modeled dense collagen. The potential for healing of cutaneous wounds and thermal burns has been related to immunomodulatory profile of Cramoll 1,4 described in other studies, including proinflammatory action in polymorphonuclear cells, induction of cytokines release, and proliferation of fibroblasts.

A lectin from* Eugenia malaccensis* seeds (EmaL) induced healing of cutaneous wounds in mice [130]. Surgical wounds produced in the skin followed by daily treatment with topical administration of EmaL reduced the intensity of inflammatory signals such as edema and hyperemia. However, higher reepithelialization with well-organized collagen fibers than control group was observed. EmaL was efficient to induce the repairing of cutaneous wounds and can be useful for therapeutic applications.

*Parkia pendula* seed lectin showed a potential healing effect on cutaneous wounds in normal and immunocompromised mice [131]. The lectin was daily topically administered in wounds produced in the dorsal region of mice. Histopathological analysis revealed edema and hyperemia during inflammatory period; no bacterium proliferation and complete wound closure were observed in normal and immunocompromised groups treated with this lectin.

Previous studies with plant lectins demonstrated their potential to stimulate the production of metalloproteinase-9 (MMP-9) that participates in different steps of wound healing, as chemoattractive factors for inflammatory cells, inductors of cytokines release, and synthesis of collagen [132]. Healing impaired is a relevant therapeutic problem in tissue repair, mainly for patients with diabetes and other diseases, which suffer from chronic wounds and require more care for cicatrization. In this context, some lectins are prohealing natural source molecules, very efficient for induction of faster reepithelialization and cicatrization.

## 5. Lectins for Drug Delivery

The therapies using chemical agents have some barriers, mainly regarding the need of increasing dosages and action of metabolism, which reduce the effectiveness of treatment. Systems for delivering drugs to a specific target may constitute interesting and effective strategies to troubleshoot these problems and minimize negative side effects [133]. The controlled deliverance techniques, such as liposomes, nanosuspensions, and bioadhesive systems, provide an adequate release rate and duration, producing the desired effect; however they have a main disadvantage of nonspecificity to substrate [134]. On the other hand, the lectin-mediated bioadhesion constitutes specific establishing interactions with receptor-like structures in cell membrane, binding directly to target cells [135].

**Figure 3:**
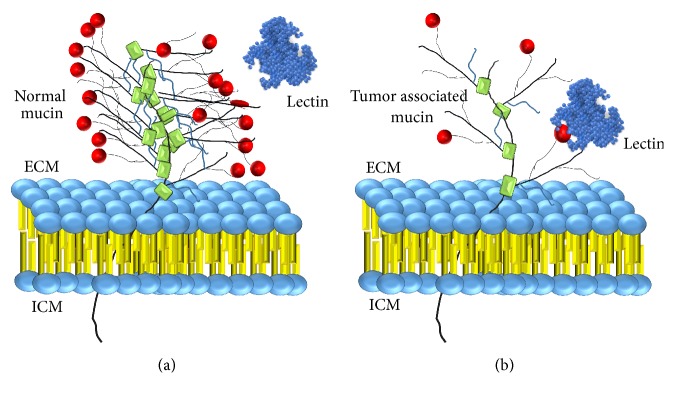
Differential interaction of lectins with cells expressing diverse glycosylation pattern. Mucins constitute a glycosylated protein family with high molecular weight, expressed by epithelial tissues. In normal cells (a) the mucin is extensively glycosylated and more than 50% of its molecular mass corresponds to oligosaccharide chains, which may be difficult or impair the interaction of lectins and the carbohydrate residues from mucin. On the other hand, in its tumor counterparts (b), the mucin generally has fewer oligosaccharide side chains, which may facilitate the binding between lectins and glycosylated sites. ECM = extracellular medium; ICM = intracellular medium.

**Figure 4:**
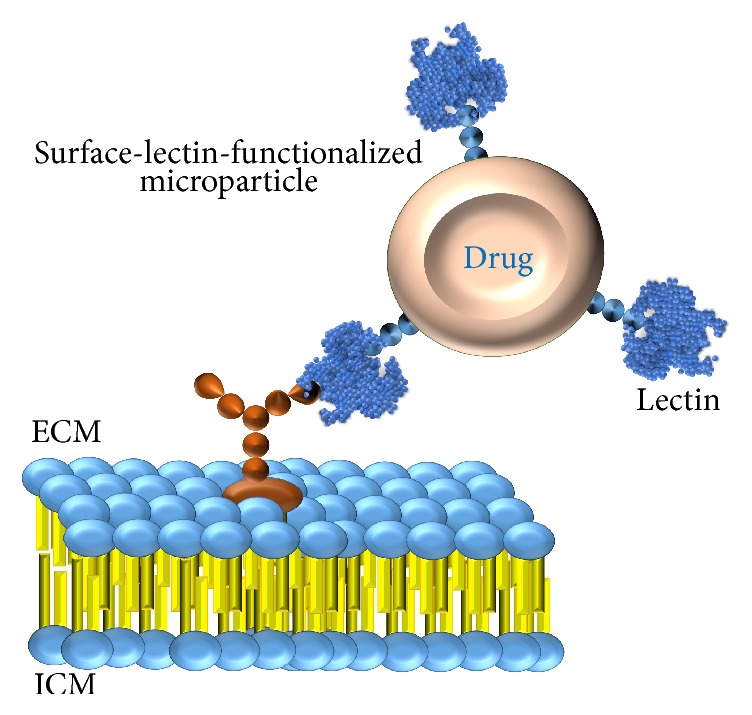
Mechanism of lectin bioadhesion. Glycoconjugates on cell surface (glycoproteins or glycolipids) can operate as lectin binding sites. ECM = extracellular medium; ICM = intracellular medium.

Since a set of cell surface proteins and lipids are glycosylated, they can operate as lectin binding sites. Different cell types generally express glycoconjugates that differ in the glycosylation patterns, as with tumor cells compared with their normal counterparts (Figure 3). In this sense, lectins can interact differently with distinct cells and may act as carriers of drugs specifically to desired cells and tissues [136]. To be a potential tool for using in drug delivery lectins should be of avid binding, low toxicity, and site-specific molecules.  Figure 4 outlines the mechanism of bioadhesion by lectins.

Lectins may interact on cell surface or be internalized via endocytosis mediated by receptors. These molecules not only allow a target specific attachment, but also can promote a drug uptake actively mediated by the cell [137]. Leong et al. [138] reported that the oral administration of insulin entrapped into surface-lectin-functionalized microparticles extended duration of the hypoglycemic effect, up to 12–24 h, in diabetic rats regarding the free insulin. Neutsch et al. [139] showed that the covalent surface modification of microparticles containing a gemcitabine derivative with wheat germ agglutinin (WGA) resulted in enhancing of binding duration on urothelial cells; bound microparticles were able to withstand the extensive washout and improved antiproliferative activity [139].

WGA covalently coupled with nanoparticles for carrying thymopentin [140]. After conjugated with nanoparticles, the lectin retained its specific carbohydrate-binding activity. In addition, the increase in WGA content on nanoparticles enhanced oral uptake of thymopentin by improving absorption of nanoparticles and protecting thymopentin against degradation. Nanoparticles coupled with WGA allowed investigation for targeted delivery of *β*-galactosidase to the intestinal mucosa in Wistar rats. Fluorometric methods showed that nanoparticles adhered to intestinal mucosa for prolonged period (6.7 h), corresponding to 6.9-fold higher than nanoparticles without lectin. Then, authors stated that WGA-nanoparticles are promising candidates for efficient mucosal drug delivery to treat lactose intolerance [141].

Liposomes modified with Con A had its degree of membrane adhesion significantly increased in comparison with liposome without lectin [133]. This adhesion followed by fusion of vesicles was also higher for Con A conjugated liposomes than for unmodified liposomes. Additionally, Con A conjugated microspheres showed high attachment rate (83.7%) in comparison with nonconjugated microspheres (16.7%) and was able to control the release of the drug amoxicillin trihydrate in simulated gastrointestinal fluids [142].

Floating-mucoadhesive microparticles containing ethylcellulose and chitosan were loaded with clarithromycin and conjugated with Con A to form a lectin-drug carrier complex against* Helicobacter pylori*. The conjugation did not interfere with the buoyancy and release of clarithromycin from microspheres using a mucus diffusion model. About 53% and 40% of drug were released from unconjugated and conjugated microspheres, respectively, within 12 h. Lectin conjugation improved mucoadhesion and interaction with porcine gastric mucin regarding unconjugated microspheres [143]. Lectins from* Pisum sativum* seeds were encapsulated in alginate microbeads for oral drug delivery against hepatocellular carcinoma; results showed that the release of lectins from microbeads depended on a variety of factors including microbead forming carriers and the amount of encapsulated lectins [144].

Acosta et al. [145] investigated the ricin B-chain (RTB) plant lectin, which corresponds to the nontoxic carbohydrate-binding B subunit of ricin AB toxin from* Ricinus communis*, as a promising carrier for human lysosomal enzymes. Authors genetically fused RTB with human *α*-L-iduronidase (IDUA), a lysosomal enzyme that degrades glycosaminoglycans. The product of this fusion RTB: IDUA retained both lectin selectivity and enzyme activity and treated human fibroblasts from normal and iduronidase-deficient individuals. The results showed that RTB: IDUA was efficiently endocytosed into human fibroblasts and able to correct the disease phenotype of mucopolysaccharidosis in fibroblasts under in vitro conditions.

Liposomes covered with* Bauhinia purpurea* agglutinin were evaluated as a drug delivery system to treat human prostate cancer. The liposomes containing the lectin were able to bind DU145-cells in mice and suppress the growth of the cells [146].

## 6. Lectins as Histochemical Markers

**Figure 5:**
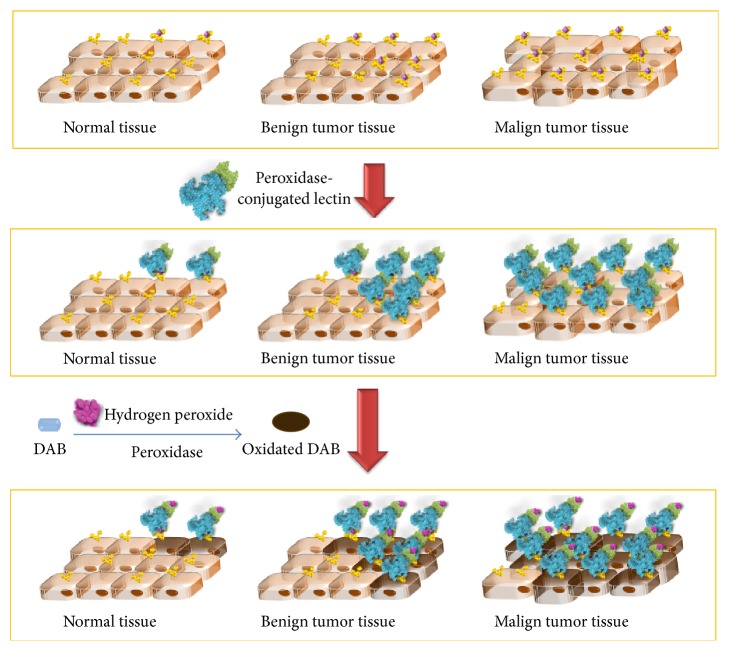
Schematic representation of lectin histochemistry using peroxidase-conjugated lectins. In this hypothetical case, the lectin binds glycan moieties more expressed on normal tissues, which suffer a modification in their structure (e.g., sialylation or fucosylation) in benign and malign tumors tissues. Thus, the lectin binding increased in transformed tissues. The DAB reagent in the presence of peroxidase and hydrogen peroxide was converted to DAB oxidized that precipitates as a brown product and allows visualization of lectin binding.

The glycan moieties covering cell surfaces are involved in many physiological and pathological processes related to cell. Disturbances in cell environment related to diseases frequently trigger changes in glycans, such as fucosylation, sialylation, abnormalities in glycan structure, and uncommon glycans [147]. Inflammation, infections, immunological disorders, and neoplasia have been associated with glycan changes [148, 149]. In this context, lectin abilities to bind carbohydrates are useful to investigate changes in the expression of glycans on cells in tissue surfaces. Histochemical analysis using conjugated lectins as potential markers for altered glycans may show differential binding patterns to normal and transformed tissues [150]. Generally, lectin histochemistry uses peroxidase-conjugated lectin followed by addition of diaminobenzidine (DAB) and hydrogen peroxidase for visualization of binding, as a hypothetical example in Figure 5. This technique has been an approach for research, diagnosis, and prognosis of human diseases signalized by altered cells in tissues, such as cancer.

Cramoll isoforms demonstrated specific binding patterns to normal and transformed human tissues. Normal and transformed (infiltrating duct carcinoma and fibroadenoma) mammary tissue sections were incubated with Cramoll 1 and Con A conjugated to peroxidase; the lectin binding patterns were visualized after interaction with DAB and hydrogen peroxidase. Cramoll 1 marked neoplastic tissues more intensely than normal tissues, similarly to Con A [151]. Cramoll 1,4 and Cramoll 3 both conjugated to peroxidase were evaluated as histochemical markers of normal, hyperplastic, and carcinoma tissue samples from human prostate; staining patterns were compared with Con A and peanut agglutinin (PNA) results [46]. Differential binding patterns were observed among normal, hyperplastic, and carcinoma tissues. Cramoll 1,4 and Con A showed more intense binding in hyperplastic than normal samples and a distinct binding pattern in carcinoma tissues, reducing the staining with the gravity of tumor. Cramoll 3 and PNA showed an increase of staining degree from normal to carcinoma tissues. Cramoll 1, Cramoll 1,4, and Cramoll 3 are potential histochemical markers for normal and transformed mammary and prostate tissues.

*Helix pomatia *agglutinin (HPA) histochemical analysis detected cancer and metastatic cells in tissues. Oligosaccharides on aggressive human breast cancer were marked by HPA histochemistry [152]. After the histochemical lectin staining, oligosaccharides were released from lectin in order to investigate some correlation between HPA binding oligosaccharides and breast cancer. A high level of monosialylated oligosaccharide HPA binding expressed in breast cancer specimens was observed, showing a positive correlation between HPA binding and aggressiveness of breast cancer. A relation between HPA binding and metastasis was reported in histochemical analysis of cutaneous malignant melanoma [153]. The lectin stained tissue sections detected primary cutaneous malignant melanoma and a positive correlation between HPA binding and metastasis. Apparently, HPA recognized N-acetylgalactosamine or N-acetylglucosamine residues on the cells and these carbohydrates have some relation with metastasis formation in malignant melanoma, being useful as histochemical marker.

In order to characterize glycosylation changes related to metastasis of breast and colon cancer cells, a histochemical analysis with HPA and selectins was performed [154]. The glycoprofiling binding of human breast and colon cancer cells, metastasizing or nonmetastasizing, was analyzed by histochemistry using lectins, among these HPA, E-selectin, and P-selectin. HPA bound metastasizing breast and colon cancer cells, while it did not bind nonmetastasizing cells. An increase of selectin ligands on metastatic colon cancer cells was observed through E-selectin binding. P-selectin binding was more intense in metastasizing breast cancer than nonmetastasizing ones. Thus, the lectin binding properties are also useful to detect metastatic cancer cells.

*Parkia pendula* lectin (PpeL) conjugated to horseradish peroxidase was evaluated as histochemical marker for characterization of meningothelial tumor tissue [155]. PpeL showed differential staining pattern that allowed identifying the meningothelial subtype. In addition, a preferential PpeL binding to cytoplasmatic glycans was observed. These results suggest the potential of PpeL as histochemical marker useful for meningothelial tumor characterization and diagnosis.

*Ulex europeus* agglutinin I (UEA-I) was indicated as candidate to prognostic marker in ovarian cancer tissues [156]. UEA-I showed a differential staining pattern related to tumor stage. Tumors with high malignancy stained by UEA-I; since this lectin is fucose-specific, it was suggested that fucose residues increase with the degree of ovarian cancer. Another study analyzed the expression of tumor-associated carbohydrate antigen to in situ breast ductal carcinoma using lectin histochemistry to detect carbohydrates [157]. The plant lectins* Griffonia simplicifolia* lectin-I (GS-I) and* Vicia villosa* agglutinin (VVA) were used for breast cancer tissue staining and results correlated to prognostic factors such as tumor size and grade as well as expression of other markers. For both lectins, more intense staining in specimens with nuclear grades II and III than nuclear grade I was observed, indicating a positive relation between expression of carbohydrate antigen GS-I and VVA-binding and more aggressive ductal carcinoma in situ.

The potential of recombinant and native frutalin, *α*-D-galactose-binding plant lectin from* Artocarpus incisa* seeds to marker human prostate tumor, was evaluated [158]. Prostate carcinoma and benign prostate hyperplasia tissues were analyzed using the lectins conjugated with anti-frutalin polyclonal antibody to react with a complex biotinylated anti-rabbit IgG and streptavidin-conjugated peroxidase, followed by the addition of DAB for visualization of lectin staining. Native frutalin showed a preferential binding for carcinoma cells compared with hyperplasic cells; recombinant frutalin marked only carcinoma cells, showing heterogeneous binding patterns. Both lectins are useful for histochemical detection of prostate tumor.

A study with human gastric cancer demonstrated association between lectin binding and metastasis formation by lectin histochemistry [159].* Maackia amurensis* leukoagglutinin (MAL) histochemical staining was performed in order to analyze the level expression of *α* 2, 3-linked sialic acid residues in gastric cancer samples and their association with metastatic potential of one cell line. High levels of *α* 2, 3-linked sialic acid residues on gastric cancer cells were evidenced by MAL, as well as relation with potential of invasion and metastasis. MAL is an efficient histochemical marker to human gastric cancer metastasis.

Con A and UEA-I were used for histochemical analysis of parotid gland mucoepidermoid carcinoma (MEC) [160]. MEC tissues of the parotid gland previously classified as low, intermediate, and high grade were incubated with Con A and UEA-I conjugated with horseradish peroxidase. Differential binding patterns for Con A and UEA-I staining were visualized. Con A binding was observed in all grades of MEC tissues, but ductal cells of high and intermediate grades were less stained. UEA-I bound intensively MEC tissues in low grade, moderately the cells in intermediate grade, and weakly MEC cells in high grade.

Lectin histochemistry has also detected fungal species infecting human tissues binding carbohydrates of their cell wall surface. Con A, UEA-I, WGA, and PNA, conjugated with horseradish peroxidase, recognized the presence of glucose/mannose, D-galactose, L-fucose, and N-acetyl D-glucosamine on the cell wall surfaces of* Aspergillus* species in human brain and lung specimens, following the visualization with DAB and hydrogen peroxide. Specimen tissues were obtained in patient autopsy diagnosed postmortem with invasive aspergillosis. The expression of methyl-*α*-D-mannoside and N-acetyl D-glucosamine detected in* Aspergillus *species by Con A and WGA staining showed the presence of fungal structures in infected specimens of brain and lung tissues [161].

Generally, lectin histochemistry uses enzyme-lectin conjugated in order to reveal the lectin binding. Glycoconjugates present in normal and transformed tissues have also been characterized by quantum dot-lectin histochemistry [162]. Con A and UEA-I were conjugated with quantum dots to bind glycoconjugates on breast tissues in accordance with specificities (*α*-D-mannose and L-fucose residues, respectively). The results revealed a differential expression and distribution of sugar residues in normal and transformed breast tissues, showing distinct binding patterns.

Lectin histochemistry is an attractive approach to mark transformed tissues and pathological events such as metastasis and shows differential lectin binding patterns that may allow distinguishing between normal, benign, and malign tumor in various grades. Lectins employed to investigate the glycan profile in transformed tissues constitute useful tools for diagnosis and prognosis of cancer.

## 7. Lectin-Based Biosensors for Disease Detection

Many known biomarkers established to specific physio- and pathological processes are glycoprotein and glycan detectable in biological fluids and cell surface [163]. Lectin assays developed for glycan analysis attached to circulating glycoproteins or cell surfaces, allowing the detection of diseases and pathogens. Lectin-based biosensors have been developed to detect and quantify glycans [164]. These systems are based on the conversion of lectin-carbohydrate interactions into a measurable signal on a surface, allowing the measurement of biomarkers.

**Figure 6:**
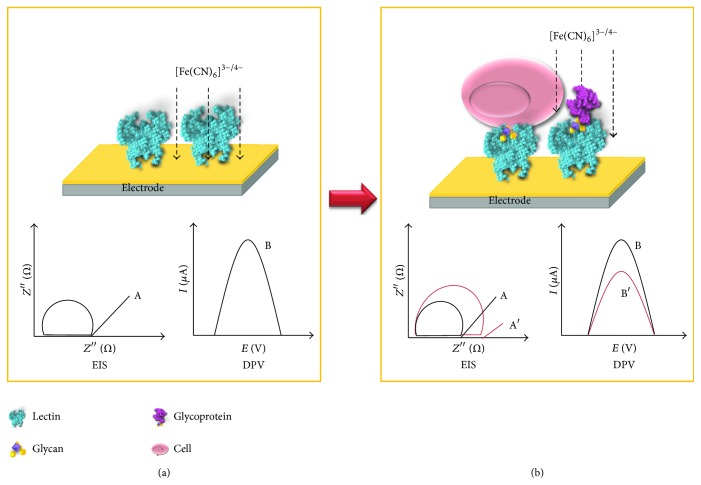
Schematic representation of lectin-modified electrode surface before (a) and after binding (b) for measurements of lectin-glycan interactions. In the electrochemical system, measurements are performed in a solution containing a redox probe (e.g., [Fe(CN)_6_]^3−/4−^); reduction or oxidation states generate electrochemical signals (charge transfer resistance for electrochemical impedance spectroscopy, EIS, and current for differential pulse voltammetry, DPV) to monitor electrode surface interactions. Before binding (a), charge transfer resistance or current signals are obtained on lectin-modified electrode surface. After binding (b), the presence of cells or glycoproteins on electrode surface generates a higher blockage for charge transfer and current signals. It is measured as an increase in the charge transfer resistance (represented by semicircle A′) for EIS response and a reduction in the current amplitude for DPV response.

When compared to other lectin techniques such as enzyme-linked lectin assay and lectin microarrays, which use labeled systems that generate color or fluorescence, biosensors can operate in a label-free mode reducing the steps and consumption of reagents. In accordance with type of signal transduction, biosensing methods can be electrochemical, optical, mass, and thermal; however, electrochemical biosensors are more attractive since they are rapid, practical, low cost, and user-friendly assays, available in distinct designs and analytical performance [164, 165]. Electrochemical biosensors have been constructed using electrodes as sensing surfaces, commonly modified with polymers and nanomaterials (carbon nanotubes, gold, magnetic nanoparticles, etc.) to improve the analytical performance and immobilization of biorecognition elements, such as lectins [164]. Techniques such as electrochemical impedance spectroscopy (EIS) and voltammetry are used to measure alterations on the electrode surface and detect interaction ligand-analyte (Figure 6). EIS measurements are based on the detection of changes in charge transfer resistance on the sensor surface after the interactions. Voltammetric techniques such as cyclic voltammetry (CV), differential pulse voltammetry (DPV), and square wave voltammetry (SWV) are based on detection of changes in the current signals generated under the application of a potential on electrode, in the presence of a redox probe [166]. Electrochemical lectin-based biosensors are more attractive as analytical tools of glycans and their application in finding pathogens and diagnosis of diseases reported through detection of biomarkers.

The specific binding affinity of Con A for glucose/mannose was explored in the development of biosensors for distinct applications. An electrochemical biosensor based on Con A was developed for nonenzymatic recognition of glucose [167]. Con A immobilized onto thionine modified electrodes established a sensing surface for specific recognition of glucose. Con A biosensor detected glucose in low concentrations in a linear range from 1.0 × 10^−6^ to 1.0 × 10^−4^ M achieved a good limit from detection of 7.5 × 10^−7^ M. The biosensor successfully determined glucose in serum samples, being a potential tool for blood glucose analysis monitored in diabetic individuals.

An electrochemical biosensor based on Con A and gold nanoparticles-modified electrode was efficient to recognize serum glycoproteins from patients infected by dengue virus [168]. Another lectin biosensor based on Con A and lipid membranes was constructed for electrochemical detection of abnormal serum glycoproteins from patients contaminated with dengue serotypes I, II, and III (DSI, DSII, and DSIII) [169]. EIS and CV revealed the interactions between Con A and glycoproteins from serum samples, showing more quantitative response to glycoproteins from DSIII. Con A recognizes serum glycoproteins related to dengue infection, being useful for determination of dengue serotypes. Another electrochemical biosensor using Con A showed potential for rapid, sensitive, and selective detection of norovirus (NoV) [170]. The biosensor was composed by a nanostructured gold electrode with Con A immobilized in order to recognize NoV. The results showed a linear relation between current signals and concentration of NoV in the range of 10^2^ and 10^6^ copies/mL, a great detection limit of 35 copies/mL, and a selectivity of 98% for NoV, being an attractive approach for sensitive and selective quantification of NoV in biological samples.

Sialylation is a common feature observed in glycans and glycoproteins in the occurrence of diseases; electrochemical lectin-based biosensors have been elaborated for determination of sialylated glycoproteins in solutions and biological samples. An ultrasensitive label-free biosensor using the sialic acid-specific lectin* Sambucus nigra* agglutinin type I (SNA-I) immobilized on a self-assembled monolayer (SAM) was developed to quantify the sialylated glycoproteins fetuin and asialofetuin [171]. EIS measurements revealed that SNA biosensor detected both glycoproteins in femtomolar level, showing a promisor application for diagnosis of diseases associated with aberrant sialylation. Another SNA-I lectin biosensor using gold nanoparticles detected sialic acid residues in fetuin and asialofetuin down to attomolar level, besides identifying changes in the sialic acid amount [172].

Biosensors, in a larger approach, were constructed based on Con A, SNA-I, and* Ricinus communis* agglutinin (RCA) on SAM-modified gold electrode in order to measure serum glycoproteins in real samples [173]. Glycoproteins were detected in femtomolar level by all three lectin biosensors. Later, they were incubated with human serum samples from healthy individuals and people diagnosed with rheumatoid arthritis (RA) for glycoprofiling of glycan patterns. Con A and RCA biosensors signals for serum from healthy individuals were weaker than signal for serum from RA patients, suggesting a lower exposition of mannose and galactose residues in serum glycans from healthy individuals. SNA biosensor showed higher signals for serum healthy individuals when compared to serum from RA patients, showing that the expression of sialic acid in serum from RA patients is reduced; it is possible to distinguish these samples due to lectin specificities. Another SNA-I biosensor was developed for discrimination of cancer-associated sialyl-Tn (STn) antigen in real samples [174]. SNA-I was immobilized onto screen-printed gold electrodes and their surfaces were incubated with serum samples from healthy individuals and patients with malignant tumors to analyze STn-expression in serum glycoproteins. EIS results revealed a differential interaction of SNA-I with STn antigens in serum glycoproteins, distinguishing between healthy and cancer samples.

Evaluation of mannose and sialic acid expression on normal and cancer cells from human lung, liver, and prostate was performed by an electrochemical lectin-based biosensor based on Con A and SNA [175]. Con A and SNA lectins were used as biorecognition element for mannose and sialic acid, respectively. The proposed biosensor could successfully detect the expression levels of specific sugars. Sialic acid was more evident in cancer cells, and mannose showed a high expression in both normal and cancer cells. The biosensor could quantify cancer cells and measure the amount of sialic acid expressed on single cell surface, being a promise approach for profiling glycan expression on cell surfaces providing an early diagnosis and treatment.

Cramoll lectin biosensors have been reported for revelation of glycoproteins in solutions and serum samples by electrochemical finding. A biosensor was developed using gold electrode modified with polyvinyl formal chloroform, Fe_3_O_4_ nanoparticles, and Cramoll for exposure of fetuin in solutions and glycoproteins from serum patients contaminated with DSI, DSII, and DSIII [176]. EIS and voltammetric measurements showed the interaction of Cramoll with fetuin and serum glycoproteins of DSI, DSII, and DSIII and a higher response to glycoproteins of DSII. Another Cramoll biosensor elaborated with gold nanoparticles, polyaniline, and Cramoll was used to identify abnormal glycoproteins of DSI, DSII, and DSIII, dengue fever, and dengue hemorrhagic fever, present in serum samples [177]. EIS and CV characterizations revealed distinct changes in the charge transfer resistance and current signals after interactions with the serum samples, being able to recognize serum glycoproteins from dengue serotypes. A greater Cramoll binding was observed to DSIII glycoproteins. A label-free Cramoll nanosensor based on assembled carboxylated carbon nanotubes and poly-L-lysine film showed differential serum glycoproteins from prostate cancer and benign prostatic hyperplasia [9]. DPV responses of nanosensor revealed that Cramoll was able to distinguish between benign and malign prostate tumor, in addition to showing a significant statistical correlation with the degree of staging prostate cancer. Another Cramoll biosensor contained self-assembled Cramoll lectin on the hybrid cysteine-gold nanoparticles-modified gold electrode and was used as a recognition interface for bacterial lipopolysaccharide (LPS) [10]. CV and EIS results expressed the selective interactions of Cramoll biosensor with LPS from* Escherichia coli*,* Serratia marcescens*,* Salmonella enterica,* and* Klebsiella pneumoniae*. Thus, Cramoll has been able to recognize bacterial LPS and serum glycoproteins, becoming a potential approach for diagnosis of diseases.

The ability of lectins to recognize glycans, in addition to the attractive analytical performance of electrochemical biosensors, has been successfully applied for detection of virus and bacteria as well as glycoprofiling of serum glycoproteins and cell surfaces. Early discovered infections and diseases can help in the diagnosis and appropriate treatment.

## 8. Lectins as Anticancer Agents

Lectins from several origins exert cytotoxic effects such as inhibition of proliferation and activation of cell death pathways, on different types of cancer cells. In addition, many anticancer lectins usually possess low cytotoxicity to nontransformed cells. This fact is probably associated with the distinct expression of glycans on surface of cancer and normal cells, allowing lectins specifically to recognize malignant cells [11, 12].

The ability of lectins to inhibit the growth of cancer cells in vitro is well documented in the literature and some examples are listed in Table 1 [13–21, 23–25, 27–29, 89, 111, 178–181]. In addition, to reduce cell proliferation, lectins may interact with receptors and other molecules present in cell surface and/or cytosol, activating cell death pathways. MCF-7 (human breast cancer) and HCT-15 (human colorectal adenocarcinoma) cells treated with a lectin isolated from* Morus alba *leaf showed morphological changes and DNA fragmentation that are characteristic of death by apoptosis, which was confirmed by the authors using staining with annexin V and acridine orange/ethidium bromide; in addition, an increased activity of caspase 3 was detected [182]. On the other hand, the* Bauhinia forficata *seed lectin induced necrosis and secondary necrosis in MCF-7 cells, with caspase 9 inhibition [21].

A mannose-binding lectin from* Clematis montana* induced apoptosis in L929 cells (murine fibro sarcoma) with activation of caspases. Authors proposed that there is a correlation between carbohydrate-binding ability and anticancer effect of this lectin since the cytotoxic activity decreased with the assay performed in presence of mannose [23]. In the same sense, Carvalho et al. [20] attributed death of NB4 (leukemia) cells promoted by* Artocarpus heterophyllus *lectin to the recognition of a trimannosyl core of* N*-glycans containing a *β*1,6-*N*-acetylglucosamine branch linked to *α*-1,6-mannose.

Mechanisms of apoptosis or necrosis induction by lectins have been studied. The treatment of MCF-7 cells with lectin from* Abelmoschus esculentus* led to increasing in expression of proapoptotic genes (caspase 3, caspase 9, and p21) as well as increased Bax/Bcl-2 ratio, being Bax a proapoptotic protein with activity inhibited by Bcl-2 [19]. Wu et al. [181] reported that* Polygonatum odoratum *rhizome lectin induced apoptosis and autophagy in A549 cells (human adenocarcinoma from alveolar basal epithelial cells) and demonstrated that this activity involves the regulation of microRNAs levels. Authors described a downregulation of the microRNA-1290, which leads to amplification of apoptosis and downregulation of Wnt pathway; in addition, the glycogen synthase kinase-3*β* was reported as a direct target of this microRNA. On the other hand, authors mentioned upregulation of microRNA-15a-3p, which mediates ROS-p53 44 pathway linked to apoptosis and autophagy.* Bothrops leucurus *venom lectin triggered necrosis in B16-F10 (murine melanoma) cells, with increase in cytosolic calcium concentration and mitochondrial superoxide generation; this lectin activated the opening of mitochondrial permeability transition pore [16]. Yang et al. [183] showed that dimerization and formation of a hydrophobic pocket in the structure of an* Agrocybe aegerita *lectin are essential for its apoptosis-inducing activity.

Lectins may also affect the adhesion ability of cancer cells. Lebecin (a C-type lectin-like protein from* Macrovipera lebetina *venom) inhibited the integrin-mediated attachment of MDA-MB-231 (human breast cancer) cells to fibronectin and fibrinogen [17];* Bauhinia forficata *seed lectin inhibited adhesion of MCF-7 cells to laminin, collagen I, and fibronectin by decreasing expression of *α*1, *α*6, and *β*1 integrin subunits [21].

As mentioned above, lectins can reach the cytosol and promote several alterations in cell physiology. A remarkable example is the lectin from Northeast China black beans, which was reported to bind HCT116 (colorectal carcinoma) cell membrane and was found in the Golgi apparatus and lysosomes within 3 h after treatment. Authors also described that this lectin caused aggregation of Golgi complex, protein accumulation in the endoplasmic reticulum, mitochondrial malformation, and membrane depolarization [29].

The several reports on the potential of lectins for cancer treatment stimulated studies at in vivo conditions using different models.* Crataeva tapia *bark lectin showed antitumor activity on sarcoma 180 (ascitic tumor) model and its safety for use in future clinical studies was indicated by the low toxicity (LD_50_ of 2,500 mg/kg) to mice [184]. Lectins isolated from fruiting bodies of* Russula lepida *and* Pleurotus citrinopileatus* also showed antitumor activity in mice, reducing in 80% and 67.3%, respectively, the growth of sarcoma 180 tumor [185, 186].

*Cratylia mollis *seed lectin encapsulated into liposomes was evaluated for in vivo antitumor activity against sarcoma 180. Shrinkage and 71% inhibition of tumor growth were detected in comparison with untreated group; encapsulation prevented lectin-damaging effects (fibrosis, necrosis, and lymphocyte infiltration) that were observed in liver and kidney of animals treated with free lectin solution [187].

*Pisum sativum *and* Momordica charantia* seed lectins showed in vitro and in vivo inhibitory effects on Ehrlich carcinoma (ascitic tumor) in mice. The growth of tumor inhibited in 63% and 75% with* P. sativum *and* M. charantia* lectins, respectively, both administered intraperitoneally at 2.8 mg/kg/day for five consecutive days. The* P. sativum *lectin caused apoptosis involving activation of caspases while the* M. charantia* lectin did not induce this cell death mechanism. The proapoptotic gene Bax was expressed intensively in cells treated with* P. sativum *lectin [28, 180]. The seed lectin from* Glycine max *caused 82.95% inhibition of Dalton's lymphoma in mice that received it through intraperitoneal injection; induction of autophagy and apoptosis, with activation of ROS production, was detected [25].

Finally, the anticancer potential of lectins also includes antimetastatic properties. Leaf lectin from* Viscum album coloratum *showed a preventive effect against lung metastasis caused by B16-BL6 and 26-M3.1 cells in mice that received 20–50 ng of lectin through intravenous administration two days before inoculation of cancer cells. This lectin also inhibited liver and spleen metastasis of L5178Y-ML25 cells when administered one day after tumor inoculation [188].

## 9. Conclusions

Lectins from diverse sources with distinct carbohydrate recognition events have important roles for many biotechnological applications and disease therapies. In vitro and in vivo uses showed that lectins have protective effects against virus and microorganisms; they are potent modulators of immune response, mitosis, proliferation, healing, drug delivery therapies, and cancer regression. Altered glycans on cells or tissue surfaces and serum samples can be located using lectin-based techniques, such as histochemistry and biosensors, detecting diseases and infection agents. Thus, this review gathers achievements attributed to lectins with focus in biotechnological/pharmacological and therapeutic applications, being a valuable resource for more studies about biological effects, pathways, and biotechnological potential of lectins. Besides, the effects described for the same or different lectins on biological systems could unravel new interpretations or insights to the field.

## Figures and Tables

**Table 1:** Sources of lectins with in vitro inhibitory effect on growth of different cancer cells.

Source	Affected cells	Reference
Fungi		
*Agrocybe aegerita *	HeLa (derived from cervical cancer cells), HL-60 (promyelocytic leukemia), SW480 (lymph node metastasis), SGC-7901, BGC-823, and MGC80-3 (gastric cancer)	[9]

Animals		
*Aristichthys nobilis *gills	HeLa	[10]
*Bothrops leucurus *venom	B16-F10 (murine skin melanoma), HEp-2 (carcinoma), K562 (chronic myelogenous leukemia), NCI-H292 (lung mucoepidermoid carcinoma)	[11, 12]
*Macrovipera lebetina *venom	MDA-MB-231 (human breast cancer)	[13]

Plants		
*Abelmoschus esculentus *seeds	MCF-7 (breast cancer)	[14]
*Amaranthus mantegazzianus *seeds	URM-106 (rat osteocarcinoma)	[15]
*Artocarpus heterophyllus *seeds	NB4 (leukemia)	[16]
*Bauhinia forficata *seeds	MCF-7	[17]
*Clematis montana *stem	L929 (murine fibrosarcoma), HepG2 (hepatocellular carcinoma), HeLa, MCF-7	[18]
*Dioscorea opposita *tubers	CNE-2 (nasopharyngeal carcinoma), MCF-7, HepG2	[19]
*Glycine max *seeds	U373MG (glioblastoma astrocytoma), HeLa, HEp-2, HepG2, MDA-MB-231	[20]
*Lotus corniculatus*	THP-1 (leukemia), HOP62 (lung adenocarcinoma)	[21]
*Microgramma vacciniifolia *rhizome	NCI-H292	[22]
*Momordica charantia *seeds	EAC (Ehrlich ascites carcinoma)	[23]
*Morus alba* leaves	HCT-15 (colorectal adenocarcinoma), MCF-7	[9]
Northeast China black beans	HCT116 (colorectal carcinoma)	[24]
*Phaseolus vulgaris cv. *extra long autumn purple bean seeds	HNE-2, CNE-1, CNE-2 (nasopharyngeal carcinoma), MCF-7, HepG2	[25]
*P. vulgaris *Chinese pinto bean seeds	HONE-1 (nasopharyngeal carcinoma)	[26]
*P. vulgaris *cv. blue tiger king seeds	HepG2	[27]
*Pisum sativum *seeds	EAC cells	[28]
*Polygonatum odoratum *	A549 (alveolar basal epithelial adenocarcinoma)	[29]
